# Interpretable analysis of social anxiety status and influencing factors among college students

**DOI:** 10.3389/fpsyt.2026.1776296

**Published:** 2026-03-16

**Authors:** Qilu Deng, Xuewen Zhang

**Affiliations:** 1 College of Sports Science, Jishou University, Jishou, Hunan, China; 2 Graduate School, St. Paul University, Tuguegarao, Cagayan, Philippines

**Keywords:** college & university students, mental health, prediction model, social anxiety, XGB

## Abstract

**Background:**

Social anxiety is prevalent among college students. Previous studies have identified various factors influencing social anxiety; however, there is limited focus on its specific determinants in the college population and a lack of clear ranking regarding their relative importance. This gap makes it challenging to implement effective intervention strategies under resource constraints. This study aims to assess the level of social anxiety among college students, rank the importance of its influencing factors, and provide essential insights for developing effective and targeted interventions.

**Methods:**

A cross-sectional study was conducted among 5,996 college students. Data were analyzed using Extreme Gradient Boosting (XGBoost) to identify and rank the importance of influencing factors.

**Results:**

The incidence of social anxiety among college students was 47.915%. The factors predicting social anxiety, ranked in descending order of importance, were: gender, grade, physical activity level, monthly household income, depression, age, and smoking.

**Conclusions:**

Social anxiety among college students should not be overlooked. Greater attention should be directed toward factors such as gender, grade, physical activity level, monthly household income, depression, age, and smoking. Educators should consider screening for social anxiety in this population and developing individualized intervention strategies tailored to students’ specific characteristics.

## Introduction

Anxiety is typically defined as a transient emotional state characterized by feelings of tension and fear, arising from an individual’s subjective perception and appraisal of threat (1). Social anxiety, in particular, is marked by a strong desire to make a positive impression on others, accompanied by persistent self-doubt regarding one’s abilities (2). According to cognitive theories of anxiety, selective attention to threat is associated with heightened anxiety, biased interpretation of social situations, and difficulties in modifying fear-related beliefs, all of which contribute to the maintenance of social anxiety (3, 4). Recent studies indicate an increasing prevalence of social anxiety among university students. For example, in South Lima, approximately 51% of students reported moderate levels of social anxiety (1); in Navi Mumbai, about 50% of students were diagnosed with anxiety disorders (5); and in China, 41.1% of students reported experiencing symptoms of anxiety (6). Social anxiety symptoms are highly prevalent among university students (2). Research indicates that even at subclinical levels, social anxiety can disrupt individuals’ psychological well-being and social functioning (7). Research indicates that social anxiety significantly impairs the fulfillment of the basic psychological need for relatedness, hinders socio-emotional development, and exerts extensive negative effects on social adjustment (8, 9). These adverse outcomes include increased feelings of loneliness and diminished quality of peer relationships. Consequently, social anxiety has emerged as a common psychological disorder in this population, representing a prevalent and concerning emotional issue that demands attention in the developmental process of contemporary university students. Extreme Gradient Boosting (XGBoost) is fundamentally based on an integrated boosting approach. It generates weak learners by optimizing a structured loss function and employs techniques such as pre-sorting and weighted quantiles to enhance algorithmic performance, prevent overfitting, and thereby improve the model’s generalization ability (10). SHAP (SHapley Additive exPlanations) values provide interpretability analysis for the model by visualizing how and to what extent each feature quantitatively contributes to predictions (11). Building on this framework, the present study applies XGBoost combined with SHAP values to explore the influencing factors of social anxiety among university students. The aim is to provide insights that can assist educators in early identification of at-risk populations and in formulating targeted measures to prevent or mitigate social anxiety in university settings.

## Methods

### Participants

A national cross-sectional survey was conducted among college students from 15 universities across China between October and November 2025. Electronic questionnaires were distributed with the assistance of academic advisors and course instructors. The first page of the questionnaire contained an informed consent statement; participants could proceed to the survey only after providing explicit consent, while those who declined were directed to an opt-out page. The anonymous and voluntary survey took approximately 10–15 minutes to complete. A total of 6,150 questionnaires were returned. After applying listwise deletion to exclude responses with missing data or uniform answering patterns, 5,996 valid cases were retained, resulting in an effective response rate of 97.495%.

### Inclusion criteria

1. Full-time college students;
2. Voluntary agreement to participate in the study.

### Exclusion criteria

1. Presence of physical disability
2. Leave of absence or absenteeism during the survey period
3. Also participate in other research projects.

### Sample size

The sample size was calculated based on the principle of at least 10 Events Per Variable (EPV (12). With 15 variables included in this study, a minimum of 150 cases were required for modeling. Considering a potential follow-up attrition rate of 10%–20% and assuming that the training set would comprise 70% of the total sample, the total sample size needed was at least 215 cases.

### Ethical considerations

The Biomedical Ethics Committee approved the study of China and was conducted in accordance with the Declaration of Helsinki. All participants voluntarily agreed to participate and signed an informed consent form, and their personal information was anonymized. They were also informed of their right to refuse participation in the study at any stage.

### Measures

#### Demographic characteristics

Gender, Age, Residence, Household Monthly Income(￥), Father’s Educational Level, Mother’s Educational Level et al.

#### The self-rating depression scale

The Self-Rating Depression Scale (SDS), developed by Zung (13), is a tool designed to measure an individual’s subjective level of depressive symptoms. This scale consists of 20 items scored on a 4-point Likert scale, ranging from “none or a little of the time” (1 point) to “most or all of the time” (4 points). Depression severity is classified as follows: scores < 50 indicate no depression, 53–62 indicate mild depression, 63–72 indicate moderate depression, and scores > 72 indicate severe depression. The Cronbach’s α coefficient for the scale in this study was 0.826.

#### Social interaction anxiety scale

Social anxiety was measured with the 15-item self-report scale developed by Leary (14). This unidimensional instrument captures the subjective propensity for social anxiety independent of observable behaviors. Each item is rated on a 5-point Likert scale (1 = “not at all” to 5 = “very consistent”), with total scores ranging from 15 to 75. A cutoff score of 45, as commonly applied in previous research (15), was used to indicate the presence of social anxiety symptoms. The scale has demonstrated established reliability and validity in Chinese university student populations. In the present study, it showed good internal consistency, with a Cronbach’s α of 0.847.

#### Physical activity rating scale

The Physical Activity Rating Scale-3 (PARS-3), originally developed by Liang Deqing (1994) (16), was used to evaluate participants’ physical activity levels over the preceding week. This instrument assesses physical activity across three domains: intensity, frequency, and duration, each rated on a 5-point scale. Intensity and frequency scores range from 1 to 5 points, while duration is scored from 0 to 4 points. A composite physical activity score (range: 0–100) is derived by multiplying the scores of the three dimensions. Higher total scores indicate more vigorous physical activity levels. In this study, the Cronbach’s α for the sample was 0.854.

#### Statistical analysis

Statistical analysis was conducted using SPSS version 27.0. Categorical data are presented as frequencies and percentages, with between-group comparisons performed using the chi-square test. A P-value of less than 0.05 was considered statistically significant. For predictive modeling, data preprocessing, transformation, and analysis were performed in Python 3.9 using the Pandas, NumPy, and XGBoost libraries. A total of 6,150 responses were collected and initially organized in Excel. Incomplete or uniformly answered records were manually excluded, resulting in 5,996 valid entries comprising 15 integer (int64) variables. No missing values were detected during Python-based processing. To balance efficiency with robustness, a staged approach incorporating univariate analysis, LASSO regression, and binary logistic regression was employed for feature selection. Statistically significant variables (P < 0.05) identified through this process were used to develop an XGBoost model for predicting the severity of social anxiety. The model integrates multiple gradient-boosted trees, with core hyperparameters including maximum tree depth (max_depth), number of trees (n_estimators), and learning rate (default: 0.1). Remaining hyperparameters were set to their default values as defined in the XGBoost library. To balance computational efficiency and predictive performance, hyperparameter tuning was performed using Grid Search CV with 5-fold cross-validation. The search ranges were as follows: max_depth from 1 to 20 and n_estimators from 5 to 50. The optimal combination of hyperparameters was selected based on accuracy on the training set. Furthermore, the SHAP framework was applied to enhance model interpretability. Feature importance rankings derived from cross-validation were used to identify an optimized subset of predictors, thereby improving both prediction accuracy and model explainability.

## Results

### General characteristics of the participants

A total of 5,996 college students were investigated in this study, including 2,813 males and 3,183 females. Among them, 47.915% of people were rated as having social anxiety. **Table 1** shows the baseline characteristics of the social anxiety group and the non-social anxiety group. Evaluate the Gender, Age, Only Child, Household Monthly Income, Father’s Educational Level and Mother’s Educational of the two groups of participants There were statistically significant differences in Level, Region, Smoking, Drinking, Grade, Depression, and Physical Activity Level (P < 0.001). See **Table 1**.

**Table 1:** General characteristics of the participants.

Variables	Total	Social anxiety		χ²	P
		No	Yes		
Gender				207.163	<0.001
Male	2813	1743	1070		
Female	3183	1380	1803		
Age(Years)				62.474	<0.001
≤18	3052	1451	1601		
19-21	2824	1586	1238		
≥22	120	86	34		
Only Child				6.794	0.009
No	4575	2340	2235		
Yes	1421	783	638		
Residence				3.258	0.071
Rural	3713	1900	1813		
Urban	2283	1223	1060		
Monthly Household Income(￥)				33.545	<0.001
≤3000	1132	592	540		
3001-6000	2573	1247	1326		
6001-9999	1354	731	623		
≥10000	937	553	384		
Father’s Educational Level				14.499	0.002
Junior high school	3095	1540	1555		
High School	1752	946	806		
University	1064	589	475		
Postgraduate	85	48	37		
Mother’s Educational Level				12.777	0.005
Junior high school	3544	1783	1761		
High School	1525	844	681		
University	874	464	410		
Postgraduate	53	32	21		
Educational Attainment				4.711	0.030
Associate Degree	1461	797	664		
Bachelor’s Degree	4535	2326	2209		
Region				13.503	0.036
North China	444	266	178		
Northeast China	1216	731	485		
East China	928	603	325		
Central China	1565	905	660		
South China	444	264	180		
Southwest China	434	257	177		
Northwest China	965	589	376		
Smoking				46.057	<0.001
No	5530	2810	2720		
Yes	466	313	153		
Drinking				10.980	<0.001
No	5073	2596	2477		
Yes	923	527	396		
Grade				115.664	<0.001
Freshman	4073	1958	2115		
Sophomore	1434	825	609		
Junior	427	310	117		
Senior	62	30	32		
Family Structure				0.002	0.966
Single-Parent Family	694	362	332		
Dual-Parent Family	5302	2761	2541		
Depression				8.671	0.003
No	2366	1288	1078		
Yes	3630	1835	1795		
Physical Activity Level				163.495	<0.001
Lightly Active	4084	1907	2177		
Moderately Active	911	538	373		
Very Active	1001	678	323		

### Screening of risk factors for social anxiety

**Figure 1:**
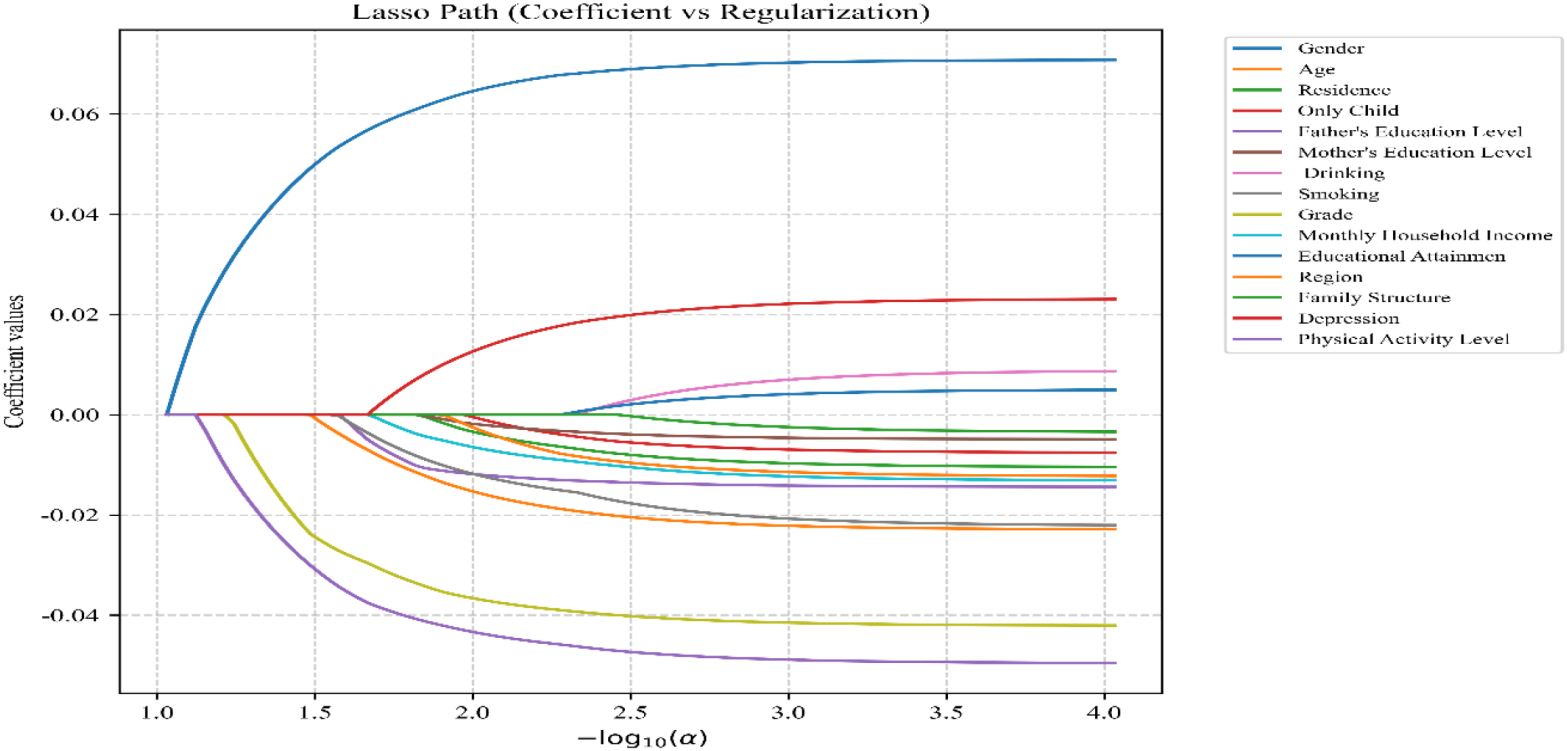
LASSO regression screening variables.

**Figure 2:**
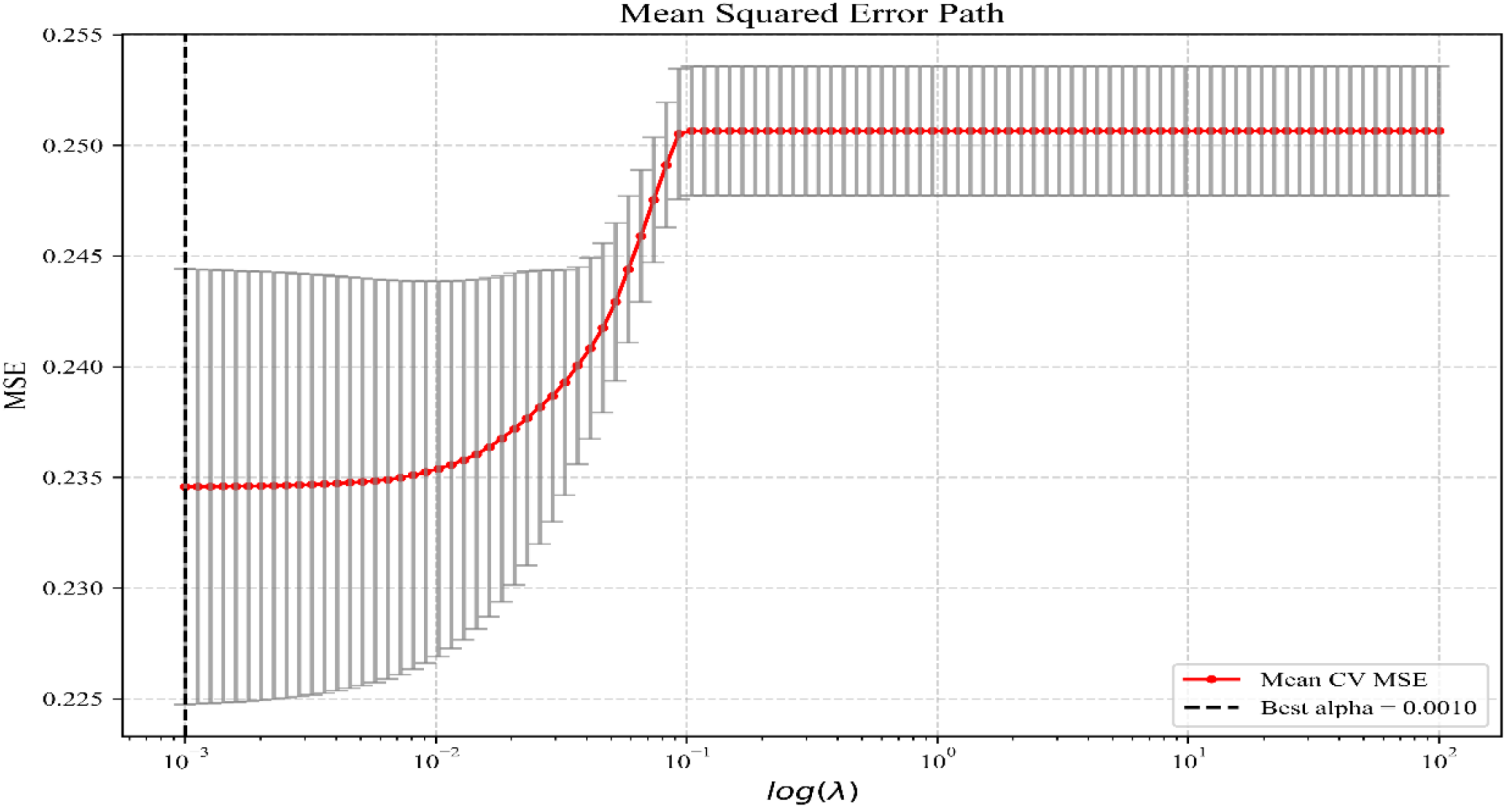
The selection of optimal λ by cross-validation.

The detailed process of LASSO regression is shown in **Figures 1**, **2**. LASSO regression determined the optimal λ to be 0.0010, and based on this, 15 predictor variables with regression coefficients ≠0 were screened out: Gender (0.139), Age (-0.039), Residence (-0.017), Only Child (-0.014), Father’s Education Level (-0.018), Mother’s Education Level (-0.006), Drinking (0.009), Smoking (-0.062), Grade(-0.062), Monthly Household Income(-0.013), Educational Attainment (0.006), Family Structure(-0.007),Region(-0.002), Depression(0.043), Physical Activity Level(-0.045) (**Table 2**).

**Table 2:** LASSO regression results of predictive variables related to social anxiety among college students.

Variables	Coefficient	Lambda. min
Gender	0.139	0.0010
Age	-0.039	
Residence	-0.017	
Only Child	-0.014	
Father’s Education Level	-0.018	
Mother’s Education Level	-0.006	
Drinking	0.009	
Smoking	-0.062	
Grade	-0.062	
Monthly Household Income	-0.013	
Educational Attainment	0.006	
Family Structure	-0.007	
Region	-0.002	
Depression	0.043	
Physical Activity Level	-0.045	

### Multivariate binary logistic regression analysis

Binary logistic regression analysis revealed that gender, age, smoking, grade, physical activity level, and monthly household income were significant risk factors for social anxiety among college students, as shown in **Table 3**.

**Table 3:** Multivariate binary logistic regression analysis of social anxiety in college students.

Risk factor	Reference factor	B	SE	Waldx^2^	P	OR	95%CI
Gender	Male						
Female		0.526	.061	74.169	<0.001	1.692	1.501-1.907
Age	≥22						
≤18		-0.135	0.066	4.209	0.040	0.873	0.768-0.994
19-21		-0.604	0.253	5.703	0.017	0.546	0.333-0.897
Residence	Urban						
Rural		-0.112	0.066	2.864	0.091	0.894	0.785-1.018
Only Child	No	-0.098	0.069	2.021	0.155	0.906	0.791-1.038
Father’s Education Level	Junior high school						
High School		-0.090	0.073	1.539	0.215	0.914	0.792-1.054
University		-0.130	0.101	1.679	0.195	0.878	0.721-1.069
Postgraduate		0.067	0.295	0.052	0.819	1.070	0.600-1.906
Mather’s Education Level	Junior high school						
High School		-0.113	0.076	2.190	0.139	0.893	0.769-1.037
University		0.021	0.110	0.035	0.851	1.021	0.824-1.265
Postgraduate		-0.167	0.367	0.208	0.648	0.846	0.412-1.735
Drinking	No	0.103	0.084	1.498	0.221	1.109	0.940-1.308
Smoking	No	-0.380	0.118	10.3665	0.001	0.684	0.542-0.862
Grade	Freshman						
Sophomore		-0.305	0.079	14.971	<0.001	0.737	0.631-0.860
Junior		-0.570	0.136	17.648	<0.001	0.566	0.434-0.738
Senior		0.303	0.299	1.027	0.311	1.354	0.754-2.432
Educational Attainment	Associate Degree						
Bachelor’s Degree		0.003	0.070	0.002	0.967	1.003	0.874-1.151
Family Structure	Dual-Parent Family						
Single-Parent Family		-0.044	0.086	0.262	0.609	0.957	0.808-1.133
Depression	No						
Yes		0.182	0.057	10.292	0.001	1.199	1.073-1.340
Physical Activity Level	Lightly Active						
Moderately Active		-0.369	0.078	22.321	<0.001	0.691	0.593-0.806
Very Active		-0.542	0.082	44.162	<0.001	0.582	0.496-0.682
Region	North China						
Northeast China		-0.009	0.113	0.006	0.940	0.991	0.794-1.238
East China		-0.216	0.119	3.318	0.069	0.805	0.638-1.017
Central China		0.091	0.110	0.694	0.405	1.096	0.884-1.358
South China		0.019	0.137	0.019	0.891	1.019	0.779-1.332
Southwest China		0.029	0.138	0.044	0.834	1.029	0.786-1.348
Northwest China		-0.047	0.117	0.162	0.688	0.954	0.758-1.200
Monthly Household Income	<3000						
3001-6000		0.192	0.075	6.503	**0.011**	1.212	1.045-1.404
6001-9999		0.004	0.088	0.003	0.960	1.004	0.845-1.194
≥10000		-0.087	0.102	0.727	0.394	0.917	0.750-1.120
-2 log-likelihood (-2LL)	7708.913						
R^2^	0.069						

### XGBoost model results

#### Feature selection

Using social anxiety (a binary outcome) among college students as the dependent variable, features including gender, age, smoking, grade, physical activity level, depression and monthly household income were included based on univariate analysis (P < 0.05).

#### Feature importance ranking

**Figure 3:**
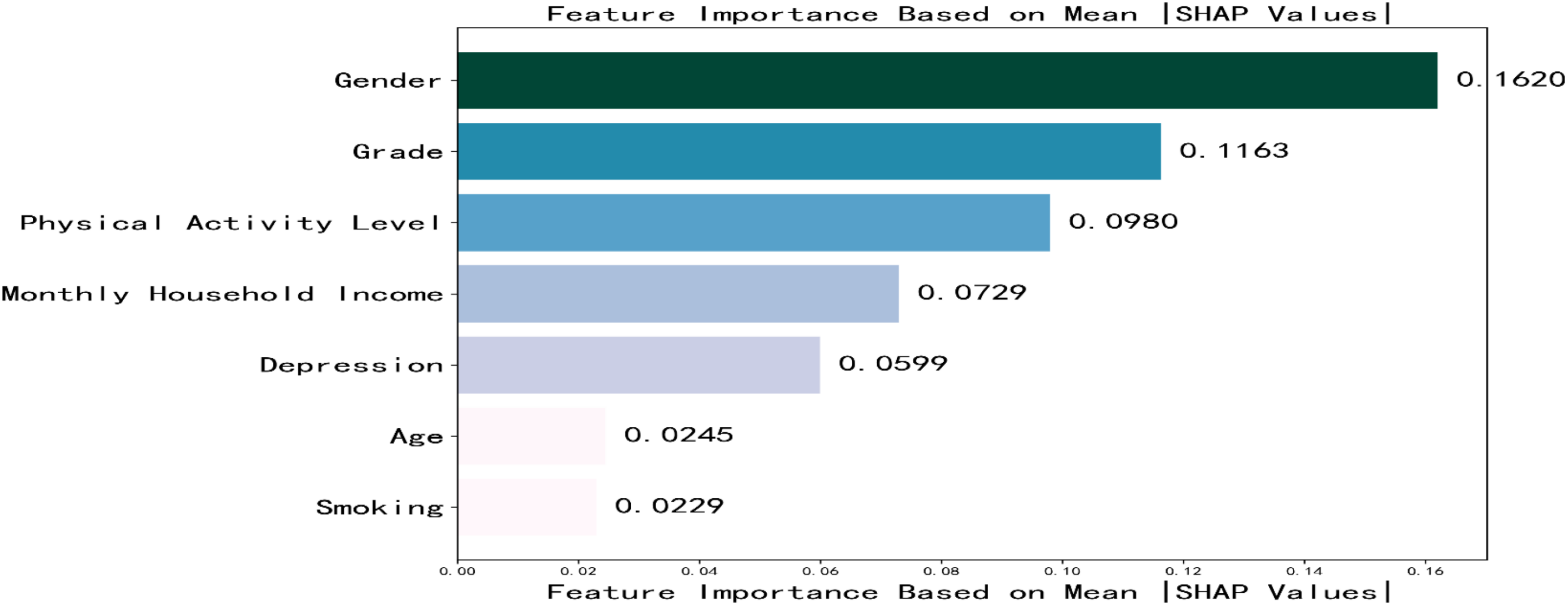
Feature importance ranking.

**Figure 4:**
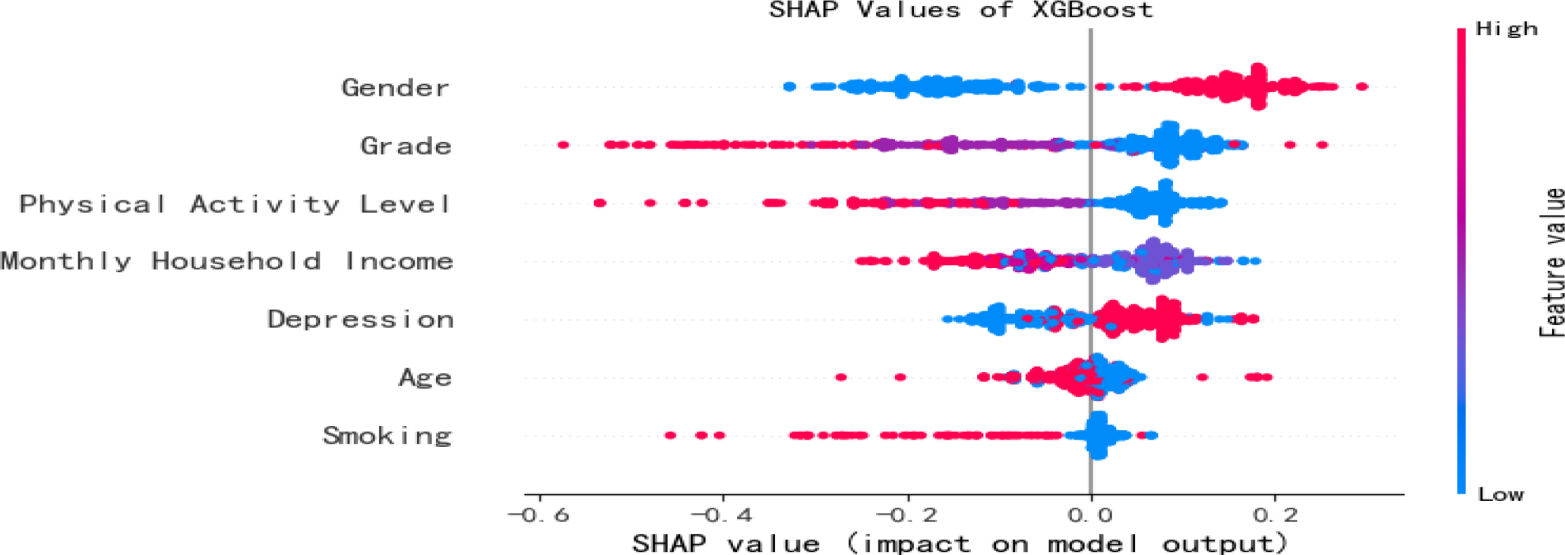
SHAP value (impact on model output).

Based on the optimal feature subset, the dataset was split into training (4,797 samples) and testing (1,199 samples) sets in an 8:2 ratio to construct an optimized XGBoost classification model. The optimal hyperparameters were determined as max_depth = 8 and n_estimators = 8. SHAP analysis was applied to elucidate the contribution of each feature to the prediction of social anxiety among college students. The SHAP feature importance bar plot (**Figure 3**) revealed the mean absolute SHAP values in descending order as follows: gender, grade, Physical activity level, monthly household income, depression, age, smoking. The SHAP feature density scatter plot (**Figure 4**) further illustrated the relationship between feature value distributions and prediction outcomes.

## Discussion

Our study revealed that the prevalence of social anxiety among college students was 47.915%, which is lower than the rate of 73.21% reported by Zhang et al (17). in a survey conducted among college students in Changsha. This discrepancy may be attributed to high heterogeneity across studies, potentially influenced by factors such as sample size, measurement instruments, and regional variations (18). Despite the lower prevalence observed in our study, the level of social anxiety remains considerably high, underscoring its widespread presence among college students. These findings further indicate that social anxiety has become a common psychological issue in this population, significantly impairing mental and physical health, and markedly reducing subjective well-being and the quality of interpersonal relationships (19). Therefore, clarifying the current status and influencing factors of social anxiety among college students, along with early detection and timely support, is of great significance for preventing the onset of social anxiety in college students and mitigating adverse outcomes associated with social anxiety symptoms. This study aimed to explore factors associated with social anxiety among college students. Using XGBoost, we assessed the relative importance of various influencing factors. The results indicated that the factors affecting social anxiety in order of importance were as follows: gender, grade, physical activity level, monthly household income, depression, age, and smoking.

Gender is a significant factor influencing social anxiety among college students. Female participants generally exhibit higher levels of interpersonal anxiety than their male counterparts (20). This discrepancy may be explained by fluctuations in sex hormones and female physiological characteristics. Hormonal variations can heighten anxiety susceptibility, when females are more prone to appearance-related anxiety compared to males. Additionally, from the perspective of social role expectation theory, women and men undergo different socialization processes and assume distinct societal responsibilities, leading to divergent psychological development. Females are often socialized to strive for approval, which may cultivate personality traits such as gentleness and introversion. Traditional societal expectations regarding gender roles further contribute to women’s heightened anxiety in interpersonal situations. This aligns with the cognitive-behavioral model of social anxiety, which posits that increased self-focused attention exacerbates social anxiety symptoms (21). Furthermore, our study identified academic year as another significant factor influencing social anxiety among college students, a finding consistent with the results reported by Nishika et al (22). Further analysis indicated that academic year ranked second in importance among all influencing factors. Notably, social anxiety levels were particularly pronounced among sophomore and junior students. This may be attributed to the escalating academic pressure and increasingly complex social relationships that accompany progression to higher grades (23), which can further exacerbate the degree of social anxiety.

Physical activity level was identified as a factor influencing social anxiety among college students, a finding consistent with previous research (24). College students with higher levels of physical activity generally demonstrate greater participation in activities, higher physical self-esteem, better social skills, and enhanced well-being (25). During physical activity, peer relationships and flow experience also play significant roles. Furthermore, studies have found that physical activity can indirectly affect social anxiety through factors such as psychological resilience (26), body image, and self-esteem (27). As noted by Si et al (28)., physical exercise promotes the release of dopamine, which elevates mood and increases energy, while also reducing adrenaline secretion. This process effectively diminishes individuals’ perception of negative emotions, thereby alleviating social anxiety among college students.

The findings of this study indicate that household monthly income is a significant factor influencing social anxiety among college students. Students from economically constrained backgrounds, while sharing similar living and academic environments with their higher-income peers, often become acutely aware of disparities in lifestyle and consumption. Their limited capacity to participate in comparable activities or achieve similar levels of satisfaction can lead to a sense of psychological disparity, which may foster feelings of inferiority and further undermine social confidence and behavioral engagement. Furthermore, lower household economic status is generally associated with limited material and socio-cultural resources from parents, and the family’s position in the socioeconomic structure may indirectly affect students’ psychological adjustment (29). Socioeconomic disparities extend beyond the material dimension and are likely to influence college students’ psychosocial adaptation and well-being through mechanisms such as unequal access to resources, restricted opportunities, and challenges related to self-identity and social valuation (30).

Depression is a significant factor influencing social anxiety among college students, a finding consistent with prior research (31). Individuals with depressive symptoms tend to exhibit an attentional bias toward negative stimuli, which often manifests as low self-confidence and poor interpersonal adjustment in social situations, thereby contributing to social avoidance (32). Furthermore, individuals affected by depression are inclined to evaluate their self-worth negatively from both their own and others’ perspectives, leading to interpersonal difficulties in real-life contexts and subsequently fostering social anxiety (32). Furthermore, this study identified smoking as a significant factor influencing social anxiety among college students, which differs from the findings reported by Lujain (33) et al. College students are at a critical stage of personal development, facing multiple challenges such as academic pressure, social adaptation, and future career planning (34). These stressors may contribute to the emergence of negative emotions such as depression and anxiety (34). Studies have found that smoking may be used as a coping mechanism for negative emotions or with the expectation that it can help alleviate emotional distress (35). Furthermore, negative emotions exert a significant influence on smoking cravings among current smokers, with higher levels of negative emotions generally associated with stronger cravings (36). Research indicates that nicotine in tobacco can stimulate the release of excitatory neurotransmitters such as serotonin, dopamine, and norepinephrine (37). Abstinence following long-term smoking may lead to dysregulation of these neurochemical systems, thereby contributing to negative emotional states such as anxiety (37). However, other studies suggest that the social context of smoking may also affect an individual’s level of anxiety. Some smokers, particularly those with lower nicotine dependence, often smoke in social settings to fulfill their desire for greater social engagement (38, 39). Therefore, the underlying mechanisms linking smoking and social anxiety warrant further investigation.

### Strengths & limitations

To the best of our knowledge, this study is the first to employ XGBoost to predict the risk of social anxiety among college students. Additionally, our survey represents a multi-center, large-scale investigation involving 5,996 college students. Nevertheless, several limitations of this study should be acknowledged. First, the cross-sectional design precludes any inference of causality among the variables. Future cohort or longitudinal studies are warranted to examine causal relationships between influencing factors and social anxiety, as well as to track its developmental trajectory over time among college students. Second, although the selected factors were derived from a review of the literature, the scope of variables considered remains limited, and other significant psychological or contextual factors may have been overlooked. Therefore, the findings should be interpreted with caution. Future research should adopt well-established theoretical frameworks and employ mixed methods to comprehensively explore the multifaceted determinants of social anxiety in this population. As new contributing factors are identified, the conclusions of this study may require further validation. Finally, while this study ranked the relative importance of various factors, it did not elucidate the potential interactions, mediating relationships, or specific pathways among the variables. Although these aspects were beyond the primary focus of the current research, future studies could address them through techniques such as path analysis or structural equation modeling.

## Conclusion

In this study, the XGBoost model was employed to analyze factors associated with social anxiety among 5,996 college students. Key factors identified included gender, grade, physical activity level, monthly household income, depression, age, and smoking. The findings suggest that educators should regularly screen for social anxiety in college students, consider implementing supportive interventions, provide timely psychological counseling, and address modifiable risk factors to mitigate the occurrence of social anxiety in this population.

## Data Availability

The original contributions presented in the study are included in the article/supplementary material. Further inquiries can be directed to the corresponding author.
